# Vitamin D receptor mediates liver ischemia and reperfusion injury by autophagy-regulated M2 macrophage polarization

**DOI:** 10.55730/1300-0152.2647

**Published:** 2023-03-21

**Authors:** Mingming FANG, Chen ZHONG

**Affiliations:** 1 Department of Neurology, Affiliated Hospital of Integrated Traditional Chinese and Western Medicine, Jiangsu Province, China; 2 Department of Neurology, Jiangsu Province Academy of Traditional Chinese Medicine, Jiangsu Province, China; 3 Hepatobiliary Center, the First Affiliated Hospital of Nanjing Medical University, Jiangsu Province, China; 4 Key Laboratory of Liver Transplantation, Chinese Academy of Medical Sciences, Jiangsu Province, China

**Keywords:** Vitamin D receptor, liver ischemia and reperfusion injury, macrophage polarization, autophagy

## Abstract

Liver ischemia and reperfusion (IR) injury is the major complication of liver-related operations. Macrophage polarization has an essential effect on the mechanism of liver IR injury. Vitamin D receptor (VDR) has been found to regulate macrophage polarization and alleviate IR injury. Nevertheless, the correlation between VDR and macrophage polarization in liver IR injury has not been clearly elucidated. VDR knockout mice and wild-type littermates underwent partial liver ischemia for 90 min and reperfusion for 6 h. RAW264.7 cells were also used to verify the influence of VDR on macrophage polarization in vitro. VDR activation could promote M2 macrophage polarization and then reduce liver injury. In contrast, VDR deficiency aggravated the liver injury by disturbing M2 macrophage polarization. Moreover, autophagy participated in the effect of VDR on M2 macrophage polarization through mediating suppressor of cytokine signaling. Therefore, VDR plays a vital influence in liver IR injury. The protective role of VDR activation in liver IR injury is related to regulate M2 macrophage polarization by autophagy.

## 1. Introduction

Liver ischemia and reperfusion (IR) injury is a common complication when patients suffer from trauma, shock, liver resection, and transplantation. It has two distinct stages: ischemia injury caused by ATP depletion and shortage of oxygen; reperfusion injury induced by an excessive inflammatory response. During liver transplantation, liver IR injury is the main reason for early graft dysfunction and can also result in acute or chronic rejection (Hirao et al., 2022).

Kupffer cells, which are resident macrophages in the liver, exert a significant effect on liver IR. Under the different microenvironment, liver macrophages that include Kupffer cells would divide into the M1 or M2 macrophage subtype. In liver IR injury, M1 macrophages aggravate the liver injury by secreting inflammatory factors and recruiting other immune cells from the circulation. However, M2 macrophages can inhibit the inflammatory response and protect the liver from IR injury (Ye et al., 2020).

Vitamin D receptor (VDR), which is a nuclear receptor activated by a natural or synthetic VDR agonist, regulates the pathophysiological function. It has been proven that VDR is involved in macrophage polarization. For instance, VDR can attenuate M1 macrophage polarization and promote M2 macrophage polarization in colitis and diabetic nephropathy (Zhang et al., 2014; Wang et al., 2017). However, VDR can also enhance M1 macrophage polarization and suppress M2 macrophage polarization (Riek et al., 2012; Nouari et al., 2016). Vitamin D and its analogue can protect against liver IR injury (Seif and Abdelwahed, 2014; Kim et al., 2017). Nevertheless, there is little clarity on the mechanism of VDR in the process of liver IR injury.

Hence, this study aims to illustrate the role of VDR in liver IR injury, uncover the mechanism of VDR, and demonstrate the relationship between VDR and M2 macrophage in liver IR injury.

## 2. Materials and methods

### 2.1. Animals

The VDR knockout (KO) mice and wild-type littermates were provided by the Research Center for Bone and Stem Cells of Nanjing Medical University. The male mice used in the experiment were 6–8 weeks old and weighed 18–22 g, housed at 23–25 °C, 12 h light/dark cycles, and 45%–60% humidity. According to the purpose of the study, all mice were divided into the following groups: (i) Sham group; (ii) IR group; (iii) IR+paricalcitol (PC) group; (iv) IR+PC+chloroquine (CQ) group; (v) IR+VDRKO group, and (6) IR+VDRKO+rapamycin (RAP) group. All the experimental procedures were in compliance with the Chinese Association of Laboratory Animal Care. The standards for animal use and care were followed by the Institution Animal Care Committee (Protocol Number: NJMU08-092).

### 2.2. Liver IR injury

Before the operation, all mice should be fed without water and food for 12 h and given heparin (100 −g/kg) by intraperitoneal injection. The microaneurysm clamp was used to block the hepatic blood. The mice were subjected to 70% hepatic ischemia for 90 min and reperfusion for 6 h. Mice in the sham group received the same operation procedure without liver ischemia injury. RAP (1 mg/ kg, MCE, Shanghai, China) and CQ (60 mg/kg, Sigma-Aldrich, St. Louis, MO, USA) were given via intraperitoneal injection 60 min before ischemia. Meanwhile, PC (1 −g/kg, MCE, Shanghai, China) was administered 15 min before ischemia through intraperitoneal injection. Carprofen (6 mg/kg) was injected intraperitoneally for analgesia in the process of mice operation. The control group was given phosphate-buffered saline. The mice were euthanized 6 h after reperfusion with overdose isoflurane inhalation. All mice were kept at 29 °C in the specially designed warm container (HTP-1500 Heat Therapy Pump, Adroit Medical Systems, USA).

### 2.3. Hepatocellular function assay

The blood from mice was preserved at 4 °C overnight and then centrifuged at 3500 rpm for 10 min. The obtained serum was kept at −80 °C. The levels of serum alanine aminotransferase (sALT) and serum aspartate aminotransferase (sAST) were evaluated by an automated chemical analyzer (Olympus Automated Chemistry Analyzer AU5400, Japan).

### 2.4. Histopathological staining

Partial liver samples were fixed with 10% neutral formaldehyde, dehydrated by ethanol, treated with xylene, and then embedded in paraffin. Liver sections were handled with a series of procedures, stained with hematoxylin and eosin (HE), and then scored as previously described (Suzuki et al., 1991).

### 2.5. Transmission electron microscopy (TEM)

Liver samples were perfused with 2.5% glutaraldehyde in sodium phosphate. Hepatic ultrathin sections were counterstained with uranyl acetate and lead citrate, and they were viewed by a transmission electron microscope (JEM1400, JEOL, Japan) at 60 kV. The AMTv image capture software (Advanced Microscopy Techniques, Inc., Danvers, MA, USA) was used to count the number of autophagic vacuoles in different areas.

### 2.6. M2 macrophage polarization in vitro

RAW264.7 cells (ATCC, Manassas, VA, USA) were cultured with M-CSF (10 ng/mL) or not for 5 days. siRNA-VDR (Santa Cruz Biotechnology, Santa Cruz, CA, USA) and PC (10 nM) were added with M-CSF at the same time. For M2 macrophage polarization, it was incubated with IL-4 (10 ng/mL) for 24 h. RAP (100 nM) or CQ (10 −M) was added with IL-4 simultaneously. The assigned groups were showed in vitro: (i) IL-4 group; (ii) PC+IL-4 group; (iii) CQ+PC+IL-4 group; (iv) siRNA-NC+IL-4 group; (v) siRNA-VDR+IL-4 group; (vi) RAP+ siRNA-VDR+IL-4 group.

### 2.7. Real-time polymerase chain reaction analysis

According to the manufacturer’s protocol, RNA was isolated from frozen liver tissues and cells with the TRIzol reagent (Invitrogen, Shanghai, China). cDNA was synthesized by using the PrimeScriptTM RT Reagent Kit with gDNA Eraser (Takara, Japan). Polymerase chain reaction (PCR) was carried out by SYBR Premix Ex TaqTM (Takara, Japan).

The primer sets were as follows: interleukin (IL)-1β (sense: GTG TTT TCC TCC TTG CCT CTG AT, antisense: GCT GCC TAA TGT CCC CTT GAA T); IL-23 (sense: TGC TGG ATT GCA GAG CAG TAA, antisense: GCA TGC AGA GAT TCC GAG AGA); IP-10 (sense: CTT GAA ATC ATC CCT GCG AGC, antisense: TAG GAC TAG CCA TCC ACT GGG); Fizz1 (sense: TGA ATA CTG ATG AGA CCA TAG AGA T, antisense: GAG TCT TCG TTA CAG TGG AGG); Ym-1 (sense: GCC AGC AGA AGC TCT CCA GAAG, antisense: GAG TAC ACA GGC AGG GGT CAA TAT); Arg-1 (sense: TTG GGA AGA CAG CAG AGG AGG T, antisense: GGT AGT CAG TCC CTG GCT TAT GG); β-actin (sense: CTA CAA TGA GCT GCG TGT GG, antisense: AAG GAA GGC TGG AAG AGT GC).

### 2.8. Western blot analysis

Proteins were extracted from liver tissues and cell lysates, and the concentration was measured through a BCA Protein Assay Kit (Thermo Fisher Scientific, Shanghai, China). The proteins were resolved by sodium dodecyl sulfate-polyacrylamide gel electrophoresis and transblotted onto polyvinylidene fluoride membranes (Millipore, USA). These membranes were blocked in nonfat dry milk (5% w/v) with Tris-buffered saline containing 0.1% Tween 20 (TBS-T) at 4 °C overnight and then incubated with primary antibodies against GAPDH (Cell Signaling Technology, Danvers, MA, USA), VDR, suppressor of cytokine signaling (SOCS) 1, and p62 (Abcam, Shanghai, China). After three washes with TBS-T, the membranes were incubated with peroxidase-conjugated secondary antibody (Cell Signaling Technology, Shanghai, China) for 1 h at room temperature. The densitometry of bands was quantified by using the Quantity One software for image analysis.

### 2.9. Statistical analysis

Data were presented as mean ± standard error of the mean (SEM) and assessed with one-way analysis of variance by using the SPSS software (Chicago, IL, USA). p < 0.05 was considered statistically significant.

## 3. Results

### 3.1. VDR involved in liver IR injury

**Figure 1:**
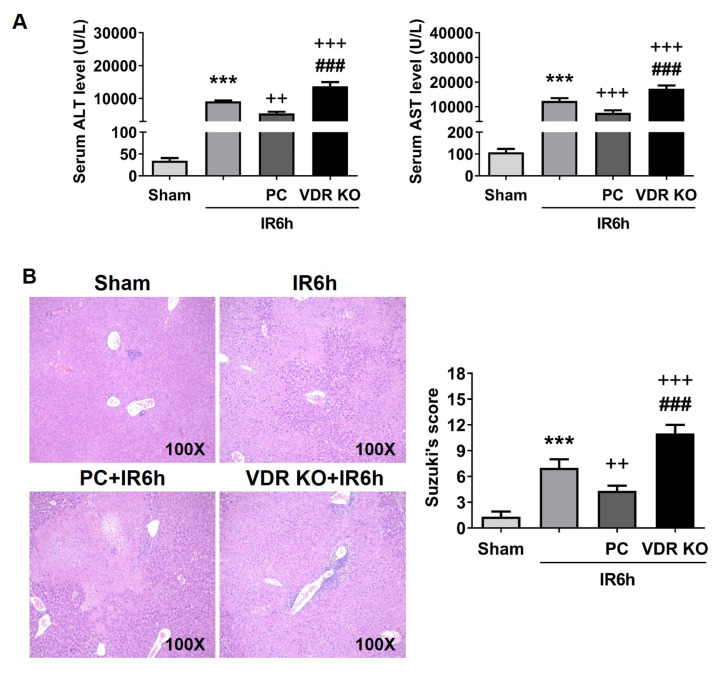
VDR activation reduced liver injury in ischemia (90 min) and reperfusion (6 h). PC was given before the operation, and VDR KO mice were applied in the study. The levels of sALT and sAST are shown in (A). Liver tissues were stained with HE and then scored in (B). Vitamin D receptor, VDR; knockout, KO; paricalcitol, PC. Data are represented mean ± SEM, n = 6 for WT mice in different groups, n = 3 for VDR deficiency mice in the VDR KO group. ^***^p < 0.001 vs. sham group; ^++^p < 0.01 and ^+++^p < 0.001 vs. IR6h group; ^###^p < 0.001 vs. PC+IR6h group.

After 6 h of reperfusion, the area of hepatocyte necrosis, Suzuki scores, and the levels of sALT and sAST were elevated in the IR group. Compared with the IR6h group, PC obviously reduced the area of hepatocyte necrosis, Suzuki scores, and the levels of sALT and sAST in the PC+IR6h group (Figure 1). The discrepancy between the IR6h group and the PC+IR6h group was statistically significant. However, the area of hepatocyte necrosis, Suzuki scores, and the levels of sALT and sAST were the highest in the VDR KO+IR6h group. There was also a significant difference between the VDR KO+IR6h group and the IR6h group.

### 3.2. VDR regulated macrophage polarization in liver IR injury

**Figure 2:**
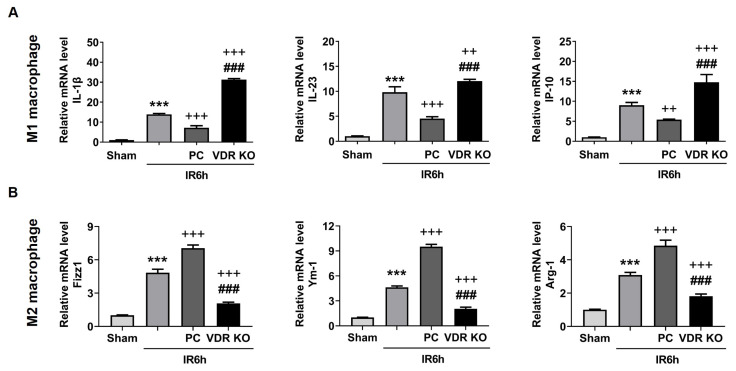
VDR activation was beneficial for M2 macrophage polarization in liver ischemia for 90 min and reperfusion for 6 h injury. The levels of M1 macrophage-associated factors (IL-1β, IL-23, and IP-10) (A) and M2 macrophage-associated factors (Fizz1, Arg-1, and Ym-1) (B) were measured. Vitamin D receptor, VDR; knockout, KO; paricalcitol, PC. Data are represented mean ± SEM, n = 6 for WT mice in different groups, n = 3 for VDR deficiency mice in the VDR KO group. ^***^p < 0.001 vs. sham group; ^++^p < 0.01 and ^+++^p < 0.001 vs. IR6h group; ^###^p < 0.001 vs. PC+IR6h group.

The discrepancy in liver injury between the PC+IR group and the VDRKO+IR group made us eager to know how VDR influenced liver injury. It is the fact that liver IR injury is the sterile inflammation, and the macrophage phenotype plays a considerable role in the process of IR. Therefore, the cytokines of M1 and M2 macrophages were assessed in the study. In the IR6h group, the level of M1 macrophage-associated factors (IL-1β, IL-23, and IP-10) and M2 macrophage-associated factors (Fizz1, Arg-1, and Ym-1) was increased in comparison with the sham group. However, in the PC+IR6h group, the level of M1 macrophage-associated factors was downregulated, but the level of M2 macrophage-associated factors was upregulated by activating VDR. The following results further proved our assumption. In the VDR KO+IR6h group, the level of M1 macrophage-associated factors was significantly upregulated, but the level of M2 macrophage-associated factors was sharply downregulated (Figure 2). Hence, we assumed that M1/M2 macrophages took part in liver IR injury and VDR mediated liver IR injury through influencing M1/M2 macrophage polarization.

### 3.3. VDR mediated M2 polarization by autophagy and SOCS1

Previous data showed that VDR had an effect on the secretion of M2 macrophage-associated factors. We postulated that VDR was related to the critical factor that regulated M2 macrophage polarization. SOCS1 has been proven to be important for M2 macrophage polarization and involved in liver IR injury (Sano et al., 2011; Whyte et al., 2011). In the study, the level of SOCS1 was increased in the IR group to repress the inflammation. However, under the influence of PC and VDR silencing, the expression of SOCS1 was the dichotomy that was consistent with the level of M2 macrophage-associated factors.

**Figure 3:**
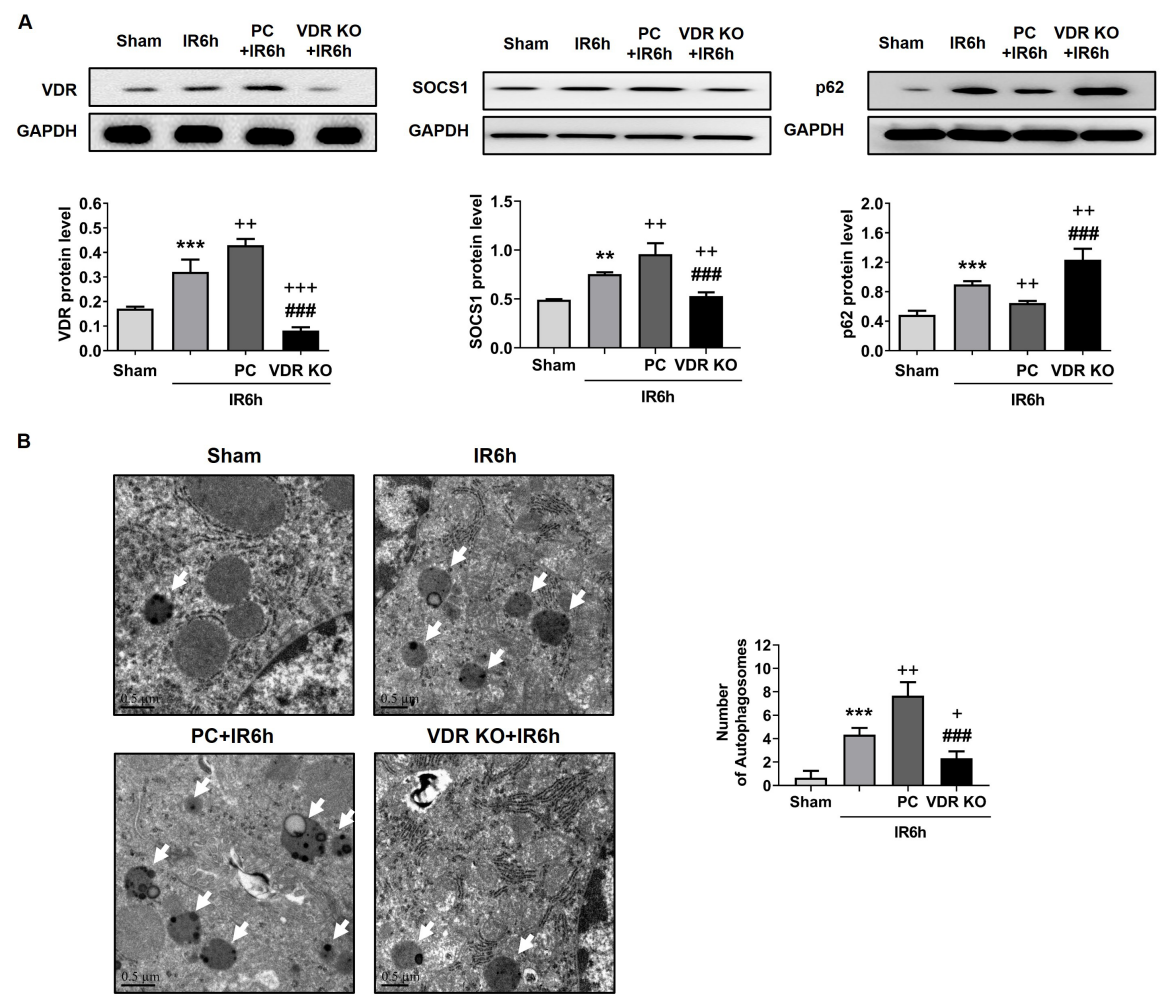
The effect of VDR on macrophage polarization was associated with SOCS1 and autophagy. After the liver suffered from ischemia and reperfusion, the expression of VDR, p62, and SOCS1 was tested by western blotting in vivo (A). The number of autophagic vacuoles was observed by TEM (B). Vitamin D receptor, VDR; knockout, KO; paricalcitol, PC. Data are represented mean ± SEM, n = 6 for WT mice in different groups, n = 3 for VDR deficiency mice in the VDR KO group. ^**^p < 0.01 and ^***^p < 0.001 vs. sham group; ^+^p < 0.05, ^++^p < 0.01 and ^+++^p < 0.001 vs. IR6h group; ^###^p < 0.001 vs. PC+IR6h group

Meanwhile, it had been illustrated that autophagy mediated macrophage polarization (Wu and Lu 2019). Hence, the expression of p62, which is closely associated with autophagy, was detected in liver IR injury. The level of p62 was increased in the VDR KO+IR6h group. However, this tendency was the opposite in the PC+IR6h group (Figure 3A). Autophagic vacuoles were also detected by TEM, and the number in different groups was statistically significant (Figure 3B). The expression of VDR was detected (Figure 3A), and it showed a significant difference in each group.

### 3.4. VDR influenced M2 macrophage polarization via autophagy in vivo

**Figure 4:**
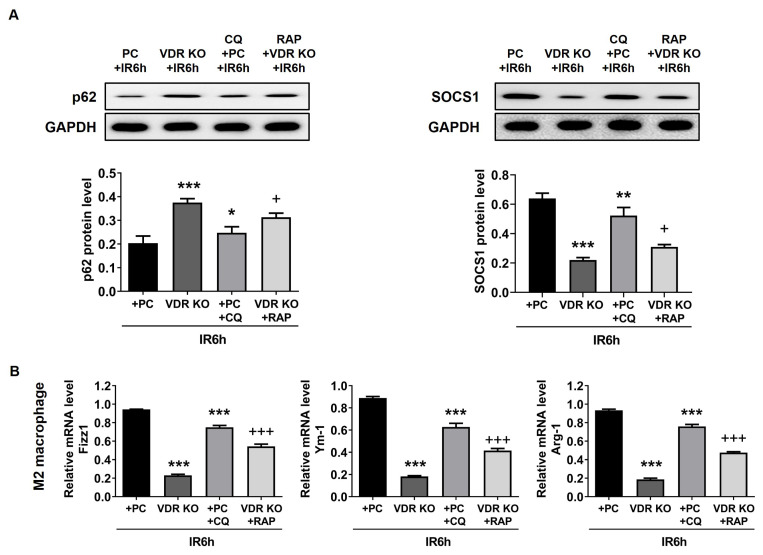
Autophagy built a bridge between VDR and SOCS1 in vivo. To uncover the relationship between VDR, autophagy, and SOCS1, RAP and CQ were used to promote or inhibit autophagy in vivo. The expression of p62 and SOCS1 was detected in (A). The level of M2 macrophage-associated factors (Fizz1, Arg-1, and Ym-1) is shown in (B). Vitamin D receptor, VDR; knockout, KO; paricalcitol, PC; rapamycin, RAP; chloroquine, CQ. Data are represented mean ± SEM, n = 6 for WT mice in different groups, n = 3 for VDR deficiency mice in the VDR KO group. ^*^p < 0.05, ^**^p < 0.01 and ^***^p < 0.001 vs. PC+IR6h group; ^+^p < 0.05 and ^+++^p < 0.001 vs. VDRKO+IR6h group.

In order to make the relationship between SOCS1 and autophagy clear in this study, the autophagy inhibitor and activator were used. In the PC+CQ+IR6h group, the expression of p62 was increased compared to the PC+IR6h group, but the level of SOCS1 and M2 macrophage-associated factors was downregulated. Compared with the VDR KO+IR6h group, the expression of p62 was decreased, and the levels of SOCS1 and M2 macrophage-associated factors were upregulated, when RAP treatment was used in the VDR KO+IR6h group (Figure 4). These data showed that VDR regulated M2 macrophage polarization through the autophagy-SOCS1 pathway.

### 3.5. VDR involved in M2 macrophage polarization in vitro

**Figure 5:**
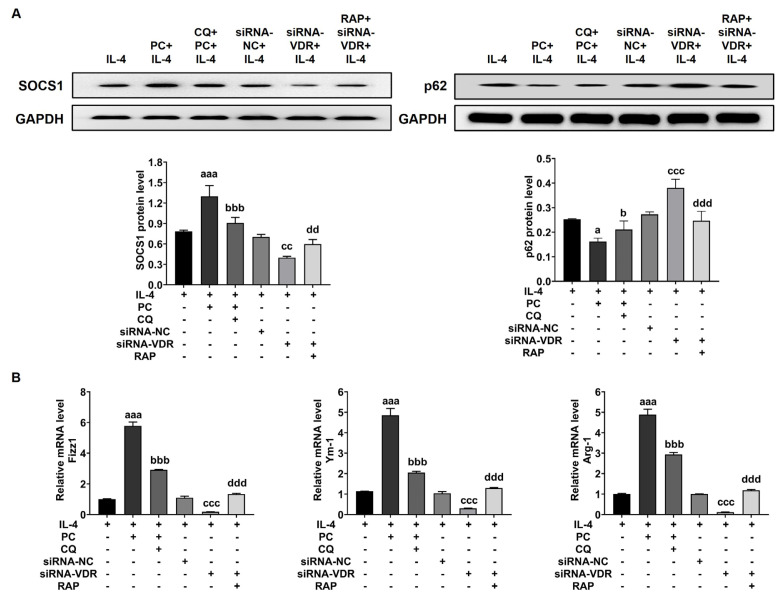
VDR regulated M2 macrophage polarization in vitro through autophagy mediating SOCS1. RAW264.7 cells by coculturing with IL-4 were polarized into the M2 macrophage subtype. The expression of SOCS1 and p62 was assessed in (A). The levels of Fizz1, Arg-1, and Ym-1 were measured in (B). Vitamin D receptor, VDR; knockout, KO; paricalcitol, PC; rapamycin, RAP; chloroquine, CQ. Data are represented by mean ± SEM. ^a^p < 0.05 and ^aaa^p < 0.001 vs. IL-4 group; ^b^p < 0.05 and ^bbb^p < 0.001 vs. PC+IL-4 group; ^cc^p < 0.01 and ^ccc^p < 0.001 vs. siRNA-NC+IL-4 group; ^dd^p < 0.01 and ^ddd^p < 0.001 vs. siRNA-VDR+ IL-4 group.

To further illustrate the effect of VDR, autophagy, and SOCS1 on M2 macrophage polarization, RAW264.3 cells were applied in our research. In the M2 macrophage polarization environment, PC was beneficial for M2 macrophage polarization and increased the expression of M2 macrophage-associated factors (Fizz1, Arg-1, and Ym-1) and SOCS1, but CQ could attenuate the effect of PC on M2 macrophage polarization, which led to a decrease in the level of M2 macrophage-associated factors (Fizz1, Arg-1, and Ym-1) and SOCS1. At the same time, the loss of function of VDR by siRNA-VDR repressed the M2 macrophage polarization and then downregulated the expression of M2 macrophage–associated factors (Fizz1, Arg-1, and Ym-1) and SOCS1; however, RAP could mitigate the inhibitory effect of the loss of function of VDR on M2 macrophage polarization. Besides, the level of p62 in different groups was also assessed and considered statistically significant (Figure 5).

## 4. Discussion

In our study, we found that PC, which is a synthetic VDR agonist, can protect the liver from IR injury, and VDR deficiency would aggravate liver injury in the process of IR. Meanwhile, the results showed that VDR affected the polarization of M1/2 macrophages. VDR deficiency caused harm to M2 macrophage polarization; however, the activation of VDR by PC was beneficial for M2 macrophage polarization. The level of M2 macrophage polarization showed a negative correlation with liver injury. Furthermore, it was shown that autophagy might be the bridge between VDR and M2 macrophage polarization. VDR activation by PC promoted autophagy and M2 macrophage polarization and decreased liver injury, but CQ could inhibit this protective role. While VDR deficiency exacerbated liver injury by inhibiting autophagy and M2 macrophage polarization, this destructive role could also be reversed by RAP.

Liver IR injury is a usual and unavoidable problem in the process of liver surgery. The mechanism of liver IR is complex, and there is no good solution to prevent this phenomenon. Innate immunity is one of the paramount factors in the mechanism of liver IR injury. Kupffer cells are liver-resident macrophages and can be polarized into M1/M2 macrophages in different microenvironments. M1 macrophage polarization is stimulated by LPS and IFN-γ. The activation of M1 macrophages will promote an inflammatory response and release a large number of inflammatory cytokines. M2 macrophage polarization is triggered by IL-4, and the activation of M2 macrophages will inhibit the inflammatory response. Our study found that M2 macrophage polarization was negative for liver injury. The mechanism of macrophage polarization is complex. However, autophagy has also been proven to regulate macrophage polarization. Although different ideas exist regarding the role of autophagy on M1/2 macrophage polarization (Shan et al., 2017; Cui et al., 2019), autophagy in our research is to promote M2 macrophage polarization, and this role could be impaired by the autophagy inhibitor CQ.

VDR is a member of the nuclear hormone receptor superfamily and plays a crucial role in mineral and bone homeostasis. Deficiency or dysfunction of VDR can result in many diseases (Wu and Sun 2011). VDR is mainly responsible for binding with and regulating the function of vitamin D and its analogues. Previous studies have illustrated that vitamin D and its analogue protect against liver IR injury (Seif and Abdelwahed 2014; Kim et al., 2017). In addition, VDR activation by PC can reduce myocardial IR (Yao et al., 2015). However, the role of VDR involved in liver IR injury or not was not known. In our study, we found that VDR activation by PC can relieve liver injury in IR, and VDR deficiency would aggravate liver injury.

VDR also participates in mediating the immune response because it is expressed in many immune cells, including macrophages, T cells, and B cells (Baeke et al., 2010). The activation of VDR in macrophages results in an immunosuppressive effect (Kiss et al., 2013). In the study, VDR activation by PC could enhance M2 macrophage polarization, inhibit inflammation, and finally alleviate liver injury. Meanwhile, VDR deficiency can deteriorate liver IR injury by disturbing M2 macrophage polarization. Previous research proved that VDR regulates inflammation through autophagy (Lu et al., 2020; Fang et al., 2021) and M1/2 macrophage polarization (Wang et al., 2017). We also found that the autophagy agonist RAP or inhibitor CQ could respectively enhance or weaken the role of VDR on M2 macrophage polarization in Figure 4. Hence, we thought VDR regulated M2 macrophage polarization by autophagy.

Autophagy is an intracellular catabolic process by lysosomes in eukaryotic cells. The function of autophagy is to maintain cellular homeostasis. It can be stimulated by physiological and pathological conditions, including protein aggregates, damaged organelles, endoplasmic reticulum stress, reactive oxygen species, and hypoxia (Chen et al., 2014). Hepatic autophagy not only maintains the balance of the liver, but also exerts a substantial effect on liver diseases (Schneider and Cuervo, 2014). In liver IR injury, enhancing autophagy is beneficial for cell survival, while long-term and excessive autophagy is harmful to cell survival. Our previous study showed that prompting autophagy can attenuate liver IR injury (Zhong et al., 2015).

Autophagy is also implicated in the process of immunity, including cell-autonomous defense, immune cell differentiation, and innate immune signaling. Furthermore, autophagy takes part in most macrophage functions, including pathogen recognition, phagocytosis, cytokine release, and inflammatory responses. In addition, autophagy mediates macrophage polarization as well (Wu and Lu 2019). It has been proven that autophagy protects against liver IR injury through regulating immunity (Wang et al., 2021). In our research, promoting or inhibiting autophagy by RAP or CQ can disturb the macrophage polarization and affect liver IR injury.

The suppressor of cytokine signaling (SOCS) family of proteins is the element of the negative feedback loop, which mediates the intensity, duration, and quality of cytokine signaling (Yoshimura et al., 2007). As negative regulators of inflammation, SOCS proteins are initiated by inflammatory cytokines and disturb cytokine signaling by targeting the JAK/STAT pathway (Alexander 2002). SOCS1 is an identified important negative regulator of inflammation (Nakagawa et al., 2002) and interferes with the proinflammatory pathways of cytokines (Fujimoto and Naka 2003). Moreover, SOCS1-knockout mice will develop symptoms of inflammatory and severe systemic autoimmune diseases (Brysha et al., 2001; Yoshimura et al., 2007). SOCS1 in macrophages and T cells has been proven to block lethal inflammation (Chong et al., 2005; Yang et al., 2008). SOCS1 is increased in M2 macrophages and determines M2 macrophage polarization (Whyte et al., 2011). SOCS1 can also protect the liver from IR injury by repressing inflammation (Sano et al., 2011). In our study, the level of SOCS1 was positively related to M2 macrophage polarization and negatively related to the liver injury. SOCS1 can induce autophagy by mammalian target of rapamycin (Chen et al., 2020), but in this research, we found that autophagy regulated SOCS1, which is consistent with its relative SOCS3 (Wan et al., 2022).

There are some limitations in our study. Firstly, the results from mice with myeloid-specific VDR gene knockout in vivo are more persuasive than that in the nonspecific VDR gene knockout mice. Meanwhile, Kupffer cells are performed to verify the results in vivo, and the effect may be better than the role of RAW 264.7 cells. Thirdly, the SOCS1 protein can be actively upregulated or downregulated in vivo; it will contribute to demonstrating the relationship between autophagy and M2 macrophage polarization.

In summary, our research illustrated that VDR activation could decrease liver injury. The protective role of VDR was related to M2 macrophage polarization by autophagy. These findings can help formulate a new clinical therapy for alleviating liver IR injury.
